# Evaluation of stress and strain on mandible caused using “All-on-Four” system from PEEK in hybrid prosthesis: finite-element analysis

**DOI:** 10.1007/s10266-022-00771-z

**Published:** 2022-11-27

**Authors:** Yomna H. Shash, Mohamed T. El-Wakad, Mohamed A. A. Eldosoky, Mohamed M. Dohiem

**Affiliations:** 1 Department of Biomedical Engineering, Helwan University, Cairo, Egypt; 2 Faculty of Engineering and Technology, Future University in Egypt, Cairo, Egypt; 3 Department of Prosthodontics, Zagazig University, Cairo, Egypt

**Keywords:** Hybrid prostheses, All on four, Titanium, Polymers, PEEK

## Abstract

Hybrid prostheses have recently been used as suitable treatment alternatives for edentulous individuals to restore the mastication mechanism. These prostheses utilize “All on four” concept, in which four implants are inserted into the jaw bone, and supported by a bar. Titanium is usually used in the fabrication of “All on four” parts due to its good mechanical properties. However, it has many drawbacks including esthetic impairment, casting issues, hypersensitivity reactions, stress shielding, and incompatibility with imaging techniques. These drawbacks have prompted researchers to find alternative materials (e.g., polymers). Recently, the new polymeric material PEEK has a major role in dentistry, due to its biocompatibility, shock-absorbing ability, and good mechanical properties. This work used the finite-element method to conduct stress–strain analysis on mandible rehabilitated with a hybrid prosthesis, using PEEK in the fabrication of “All on four” parts instead of titanium, using different densities of spongy bone. As the density of spongy bone is expected to influence the choice of “All on four” fabrication material. A 300 N vertical force was applied unilaterally, bilaterally, and anteriorly to stimulate the different mastication mechanisms. The results illustrated that PEEK material reduced the stresses and strains on bone tissues and increased the mucosal stress, compared to titanium. Consequently, this material was recommended to be used in the fabrication of “All on four” parts, especially in the low-density model. However, further research on PEEK implants and abutments is required in near future.

## Introduction

Implant-supported hybrid prostheses have recently been used as appropriate treatment options for edentulous patients to restore the mastication mechanism and the quality of life [1]. The location of the mandibular canal or the anatomic limitations of the residual alveolar bone due to resorption can cause difficulties in the insertion of dental implants. Consequently, “All on four” technique has been used to solve these difficulties and proved successful in most clinical studies [2, 3]. In “All on four” technique, two anterior vertical implants and two tilted posterior implants are inserted into the edentulous jaw. The tilting of implants reduces the cantilever length and increases the implant–bone contact, decreasing peri-implant bone stress [4]. A solid bar (framework) is attached to the four implants to support them, enhance the stress distribution and reduce the stresses generated on bone and mucosa. Finally, the acrylic teeth are arranged on the bar and secured with acrylic material [4].

Titanium has been used in the fabrication of “All on four” parts, due to its durability, biocompatibility, and excellent mechanical properties. However, it has many issues, including esthetic impairment, casting problems, metallic taste, hypersensitivity reactions, and incompatibility with imaging techniques [5]. In the fabrication of implants, titanium implant may induce a stress-shielding effect and hence implant and bone loss, because of its high elastic modulus (110 GPa) compared to bone (14 GPa) [5, 6]. Furthermore, other reasons for titanium implant failure are related to hypersensitivity reactions (e.g., erythema, urticaria, swelling, pain, eczema, and necrosis) and peri-implantitis-related surface deterioration [7–10]. In addition, the corrosion of titanium, as well as, the release of titanium ions, triggers an immunological response and hence additional negative effects [11].

In the fabrication of abutments, one major limitation of titanium abutments is their esthetics; the grey color may be visible through the restorative material or the surrounding tissues [5]. Other limitations are related to the hypersensitivity reactions and the formation of biofilm [12]. The protocol bar in “All on four” prosthesis is also made of rigid metallic materials such as titanium. As rigid bars are expected to transfer fewer stresses to the substructures, avoiding prosthesis failure [13]. However, contradictory results are seen in studies evaluating load transferred using rigid materials [14, 15].

The limitations of metals with patients’ desire to use non-metallic prostheses have encouraged the researchers to use alternative materials. Polymers have recently been introduced in dentistry in the fabrication of implants, abutments, screws, scaffolds, bridges, crowns, orthodontic wires, and removable and fixed prostheses, among those, polyether ether ketone (PEEK) [16, 17].

PEEK polymer, as a biological engineering material, has excellent biological, mechanical, and chemical properties. It has many advantages including high thermal stability (melting point ~ 334–343℃), high toughness, excellent fatigue and creep resistance, high erosion resistance, ease of fabrication and formation, excellent self-lubricating, compatibility with imaging techniques (not visible during CT and MRI scans), excellent sterilization performance and biocompatibility in vivo and in vitro [18, 19]. In addition, this material is non-toxic and inert, besides having low weight (density ~ 1.32 g/cm^3^) and a good esthetic appearance [19, 20]. The mechanical properties of PEEK can be enhanced to meet the requirements of diverse dental applications by the addition of different materials including fibers and fillers, or by surface treatment [16, 20–22].

In vivo, as an orthopedic implant, PEEK material possesses biomechanical properties that are close to human bone. This reduces the risk of bone resorption and osteolysis caused by the stress-shielding effect of implants [22, 23]. In addition, PEEK material has a compound structure, which enables better distribution of masticatory forces around the implant when compared to titanium [24]. PEEK material is also suitable for the fabrication of abutments since it has good mechanical properties and produces fewer biofilm when compared to titanium abutments [25]. Due to its low elastic modulus and high shock-absorbing ability, PEEK material is expected to evenly distribute the stresses generated during mastication on implants and bone, when used as a superstructure framework (bar), according to some studies [15, 20, 26]. Hence, PEEK material is expected to replace titanium in the fabrication of “All on four” parts.

The primary stability of fixed prosthesis is influenced by implant geometry, surgical techniques, and the quality of bone (e.g., the density of spongy and cortical bones) [27]. Bone loss is a common process subsequent the tooth loss, affecting the mandible more times than the maxilla. Besides, the spongy bone is more profoundly subjected to bone loss than the cortical bone [28]. In bone loss, bone resorption is faster than bone formation, reducing bone density and thus its strength. For spongy bone, from CT scan, 150 and 400–500 Hounsfield units [HU] are described as low and normal densities, respectively [29, 30].

In dentistry, the finite-element method (FEM) is a numerical method for analyzing the stresses and strains in the structures of any given geometry. It offers several advantages over other methods, including the precise illustration of complex geometries, the ability to model repair, and the extraction of internal stresses and strains [31]. In this research, the finite-element method was used to conduct in vitro stress–strain analysis on edentulism mandible rehabilitated with a hybrid prosthesis, using new polymeric material (PEEK) in the fabrication of “All on four” parts in place of titanium, using different densities of spongy bone. The density of spongy bone was expected to influence the choice of “All on four” fabrication material. The null hypothesis predicted that PEEK material would exhibit the lowest stresses and strains in bone tissues and represent the best scenario, in contrast to titanium.

## Materials and methods

### Designing the finite-element model

**Fig. 1:**
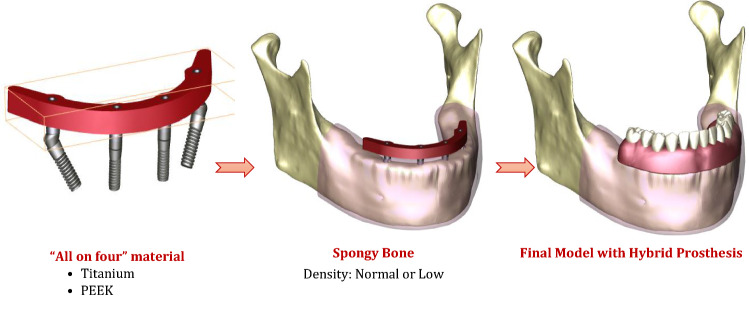
Model parts

In this section, each part was modeled separately and then all parts were gathered to construct the final model. A 3D model of an edentulous mandible was downloaded from the website (BodyParts3D/Anatomography, Life Sciences Integrated Database Center, Japan) as an OBJ file [32]. Using Solidworks software (Solidworks, Version 21, Massachusetts, USA), the mandible model was converted to solid, improved, and repaired. The mandible was segmented into 2-mm-thick cortical bone and inner volume from spongy bone, and covered with 2-mm-thick mucosa, as presented in Fig. 1.

Following the “All on four” concept, four threaded implants with their multi-unit abutments were modeled and inserted into the mandible model (Fig. 1). The dimensions were imported from the catalog of Zimmer (Zimmer, Biomet Dental, Palm Beach Gardens, USA [33]) with fine details about the shape and size. Two anterior implants (3.7 mm diameter and 10 mm length) were placed straight in the position of the lateral incisors, with straight abutments (2 mm cuff height). Two other implants (4.1 mm diameter and 11.5 mm length) were placed posteriorly with a 30° inclination angle in the position of second premolars, with angled abutments (2 mm/4 mm cuff height). Four sleeves (copings), with screws, were fitted over the straight and angled multi-unit abutments. Then, the prosthetic bar was modeled as a solid horseshoe with 5 mm width, 5.5 mm thickness, and 10 mm cantilever length, which corresponds to the configuration of the mandible [34]. Twelve acrylic teeth were arranged on the bar and secured with acrylic material (PMMA). Finally, the hybrid prosthesis had a height of 15 mm from the surface of the mucosa.

**Fig. 2:**
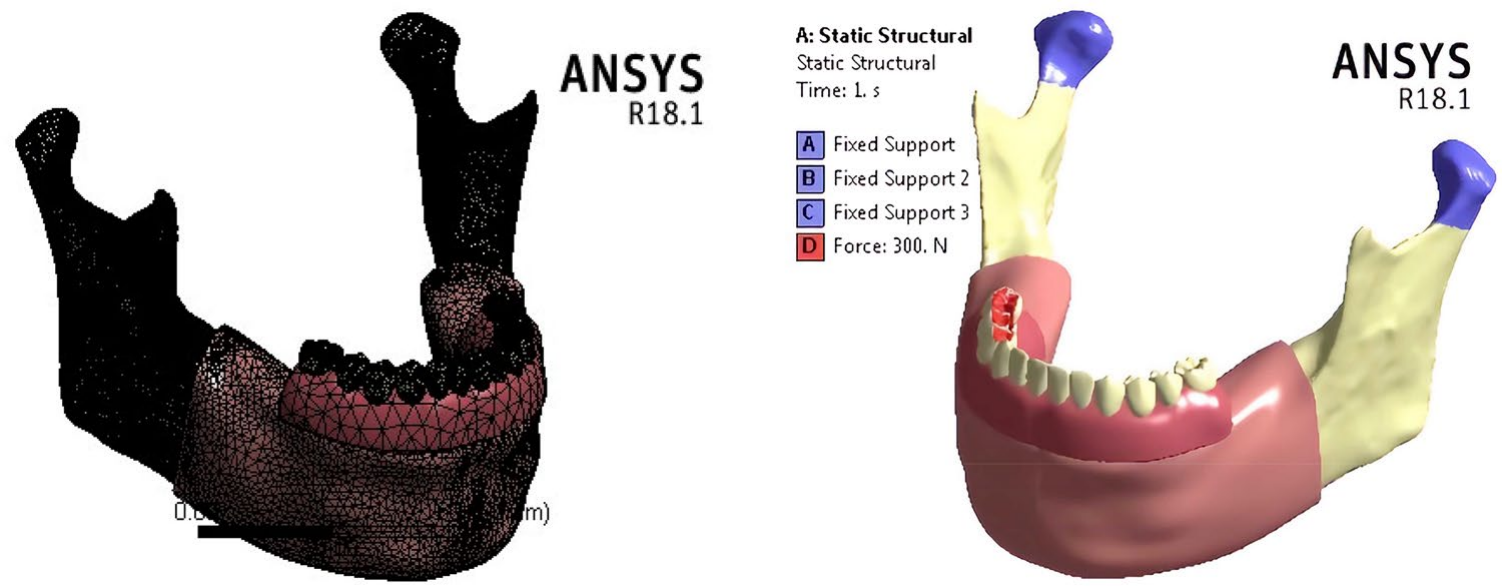
**a** Meshing. **b** Uni-lateral loading and constrains

Utilizing the Ansys software (Ansys, Version 18.0, Canonsburg, USA), the finite-element model was constructed using the “adaptive” function with a “fine” element size. The final model had approximately 434,577 elements and 760885 nodes (Fig. 2a).

### Defining the material properties

Mandible bone has been stimulated with isotropic properties because a complex model has been constructed, to reduce the computational times [35]. The values of elastic modulus of the spongy and cortical bones with normal densities have been often assumed to be 1.37 and 13.7 GPa, respectively. For spongy bone, an elastic modulus of 259 MPa has been used to stimulate the low-density case, based on previous studies [29, 30]. The prosthetic parts (bar, implants, abutments, and copings), in “All on four” prosthesis, were stimulated with two materials (Titanium and PEEK). The denture base and the artificial teeth were from acrylic PMMA. The properties of all parts are presented in Table 1 [36–38].

**Table 1:** The properties of materials

	Elastic modulus (GPa)	Poisson ratio
Cortical bone	13.7	0.3
Spongy bone		
Normal density	1.37	0.3
Low density	0.259	0.3
Mucosa	0.005	0.4
PMMA	5	0.37
“All on four” parts		
Titanium	114	0.33
PEEK	3.5	0.40

### Loading and constraint conditions

In fixed prostheses, following “All on four” concept, the average force was nearly 200–300 N for the premolars and molars region, and 100–300 N for the incisors [39]. In this research, a 300 N vertical load was applied unilaterally, bilaterally, and anteriorly to stimulate the different mastication mechanisms. In unilateral mastication, the force was distributed on the three posterior teeth (Fig. 2b), while the force was distributed on the four incisors in anterior mastication. To prevent the displacement and rotation of the model during the force application, the nodes of condyles and the inferior border of the mandible were constrained in all directions [40].

### Analysis of stresses and strains

In Ansys software, analyses were performed to compute the von Mises stresses produced on each part, in the two models (normal and low densities of spongy bone), using two “All on four” materials (Titanium and PEEK). The von Mises stress is a value used to predict the yielding/failure of materials (especially ductile materials) under complex loading. This stress value is expressed by six components to specify the state of stress at a point (Eq. 1) [41, 42]:

1 $${\upsigma }_{\mathrm{VM}}=\sqrt{\frac{{\left({\upsigma }_{X}-{\upsigma }_{Y}\right)}^{2}+{\left({\upsigma }_{Y}-{\upsigma }_{Z}\right)}^{2}+{\left({\upsigma }_{Z}-{\upsigma }_{X}\right)}^{2}}{2}+3\left({\uptau }_{XY}^{2}+{\uptau }_{YZ}^{2}+{\uptau }_{ZX}^{2}\right)}$$

${\upsigma }_{X} ,{\upsigma }_{Y}$ and ${\upsigma }_{Z}$ are the normal stresses in *X*, *Y* and *Z* directions. ${\uptau }_{XY} ,{\uptau }_{YZ}$ and ${\uptau }_{ZX}$ are the shear stresses in *X*, *Y* and *Z* directions.

For cortical and spongy bones, due to their brittle and ductile properties, the maximum (tensile) and minimum (compressive) principal stresses and strains were extracted to evaluate the yielding/failure behavior of each bone tissue.

## Results

### Max von Mises stresses

In this section, “All on four” parts were stimulated with two materials (PEEK and titanium), in different bone models (normal and low densities of spongy bone). To investigate the influence of using PEEK in the fabrication of “All on four” parts instead of titanium, and reveal its effects on the stresses transferred to bone, mucosa, and other prosthetics parts.

In the normal-bone density model as shown in Table 2, the stresses on most parts were reduced using PEEK, in contrast, the stresses on acrylic denture and mucosa were increased. Under unilateral force, the max von Mises stresses on bar, copings, screws, abutments, and implants were reduced by 38.68, 41.47, 48.54, 50.62, and 82.82%, respectively. The max stress on acrylic denture was increased by 20.57%, and the max stress on mucosa was nearly tripled. As a consequence, the max stresses on cortical and spongy bones were reduced by 12.98 and 25.69%, respectively. Under bilateral force, the max von Mises stresses on bar, copings, screws, abutments, and implants were reduced by 41.32, 50.8,51.08,53, and 82.81%, respectively, using PEEK. The max stresses on acrylic denture and mucosa were increased by 18.81 and 266%. Consequently, the max stresses on cortical and spongy bones were reduced by 21.13 and 34.61%, respectively.

**Table 2:** Max von Mises stresses (MPa) on all parts, using titanium and PEEK in “All on four”, in the normal-density model

	ALL-4	Denture base and teeth	Bar	Copings	Screws	Abuts	Implants	Mucosa	Cortical bone	Spongy bone
Uni-lateral	Ti	18.536	102.24	90.50	72.73	180.77	63.13	0.071	35.82	3.97
	PEEK	22.35	62.69	52.97	37.42	89.26	10.84	0.314	31.17	2.95
Bi-lateral	Ti	18.457	104.24	96.39	73.60	164.92	64.01	0.0867	34.77	4.16
	PEEK	21.93	61.16	47.35	36.00	77.50	11.00	0.318	27.42	2.72
Anterior	Ti	29.62	50.55	71.41	44.67	95.12	60.00	0.0476	35.56	3.95
	PEEK	32.42	18.17	29.29	23.69	27.66	14.65	0.194	28.76	3.09

Under anterior force, using PEEK, the max von Mises stresses on bar, copings, screws, abutments, and implants were reduced by 64.05, 58.98, 46.96, 70.92, and 75.58%, respectively. The max stress on acrylic denture was increased by 9.45%, and the max stress on mucosa was nearly tripled. The max stresses on cortical and spongy bones were reduced by 19.12 and 21.77%, respectively.

**Fig. 3:**
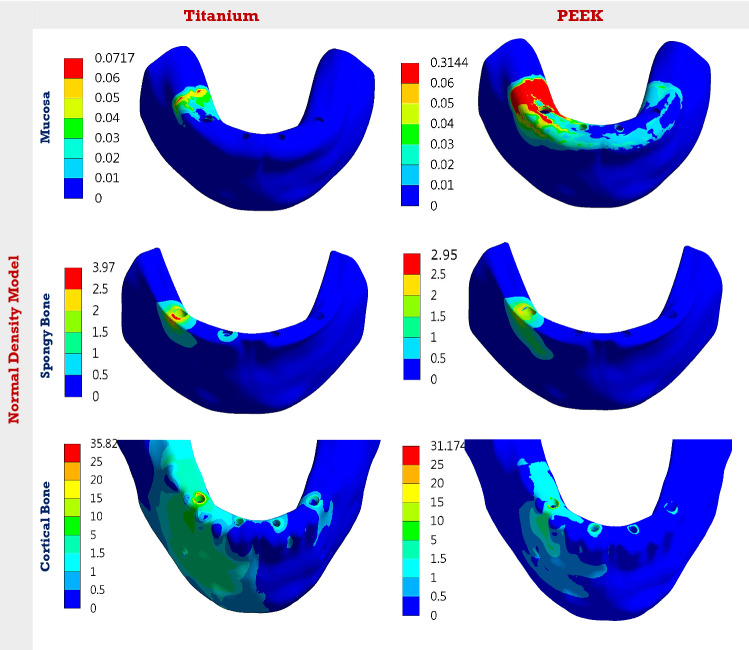
The distribution of von Mises stresses (MPa) on the mucosa, cortical and spongy bones, under unilateral force. Using Ti and PEEK. In the normal-density model

**Fig. 4:**
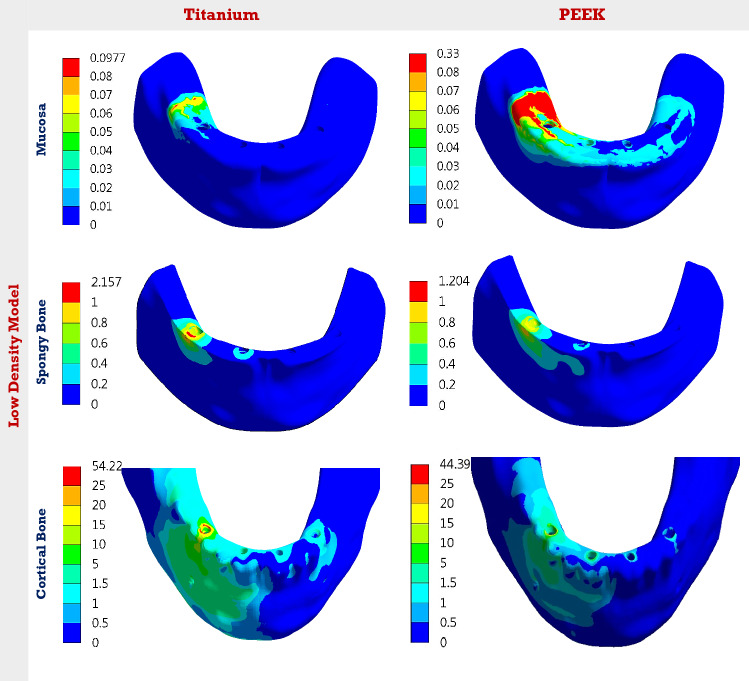
The distribution of von Mises stresses (MPa) on the mucosa, cortical and spongy bones, under unilateral force. Using Ti and PEEK. In the low-density model

In the low-density model (Table 3), the max von Mises stresses on bar, copings, screws, abutments, and implants were reduced by 43.09, 47.23, 50.87, 59.92, and 80.42% under unilateral force using PEEK. The reductions were 44.56, 51.33, 53.25, 60.37, and 81.47% under bilateral force, and 61.27, 63.78, 50.35, 74.22, and 75.16% under anterior force. The max stress on acrylic denture was increased by 20.51, 18.76, and 6.27% under unilateral, bilateral, and anterior forces, respectively, while the max stress on mucosa was nearly doubled. For cortical bone, the max stress was reduced by 18.13, 16.82, and 23.75% under unilateral, bilateral, and anterior forces. For spongy bone, the max stress was reduced by 44.18, 51.29, and 38.94%, respectively. Figures 3 and 4 illustrate the distribution of von Mises stresses on the mucosa, cortical and spongy bones under unilateral force, using titanium and PEEK, in normal and low densities models. It was clear that the use of PEEK material reduced the stresses on cortical and spongy bones, besides increasing the stress on the mucosa.

**Table 3:** Max von Mises stresses (MPa) on all parts, using titanium and PEEK in “All on four”, in the low-density model

	ALL-4	Denture base and teeth	Bar	Copings	Screws	Abuts	Implants	Mucosa	Cortical bone	Spongy bone
Uni-lateral	Ti	18.38	109.35	101.00	75.74	219.58	53.53	0.097	54.22	2.15
	PEEK	22.15	62.230	53.29	37.21	88.00	10.48	0.330	44.39	1.20
Bi-lateral	Ti	18.28	109.81	96.58	76.39	194.31	56.46	0.110	46.60	2.32
	PEEK	21.71	60.870	47.00	35.71	77.00	10.46	0.331	38.76	1.13
Anterior	Ti	29.50	46.789	80.84	47.40	107.63	59.07	0.0577	50.90	1.90
	PEEK	31.35	18.120	29.28	23.53	27.74	14.67	0.204	38.81	1.16

**Fig. 5:**
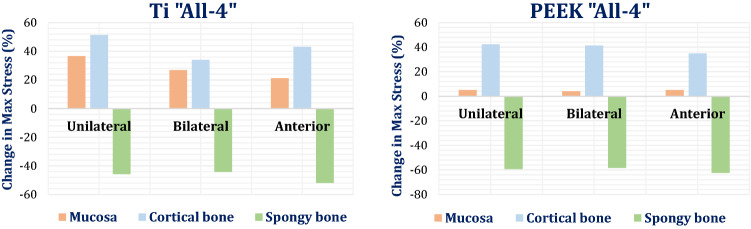
The change in max von Mises stresses (%) of the mucosa, cortical and spongy bones, in the low-density model, compared to the normal-density model. Using Ti and PEEK

Comparisons between normal and low densities models are presented in Fig. 5. Figure 5 illustrates the change in max von Mises stresses of the mucosa, cortical and spongy bones in the low-density model, compared to the normal-density model, using titanium and PEEK “All on four”. In the low-density model, using titanium, the max von Mises stress on spongy bone was reduced by 45.84, 44.23, and 51.89% under unilateral, bilateral, and anterior forces, compared to the normal-density model. Therefore, the max stress on cortical bone was increased by 51.36, 34.02, and 43.13%, respectively, and the max stress on mucosa was increased by 36.61, 26.87, and 21.21% compared to the normal-density model. Using PEEK, in the low-density model (Fig. 5), the max stress on spongy bone was reduced by 59.32, 58.45, and 62.45% under unilateral, bilateral, and anterior forces, respectively, compared to the normal-density model. Consequently, the max stress on cortical bone was increased by 42.41, 41.35, and 34.94%, respectively. Moreover, the max stress on mucosa was increased by 5.09, 4.08, and 5.15%, respectively.

### Peak max and min principal stresses and strains

**Fig. 6:**
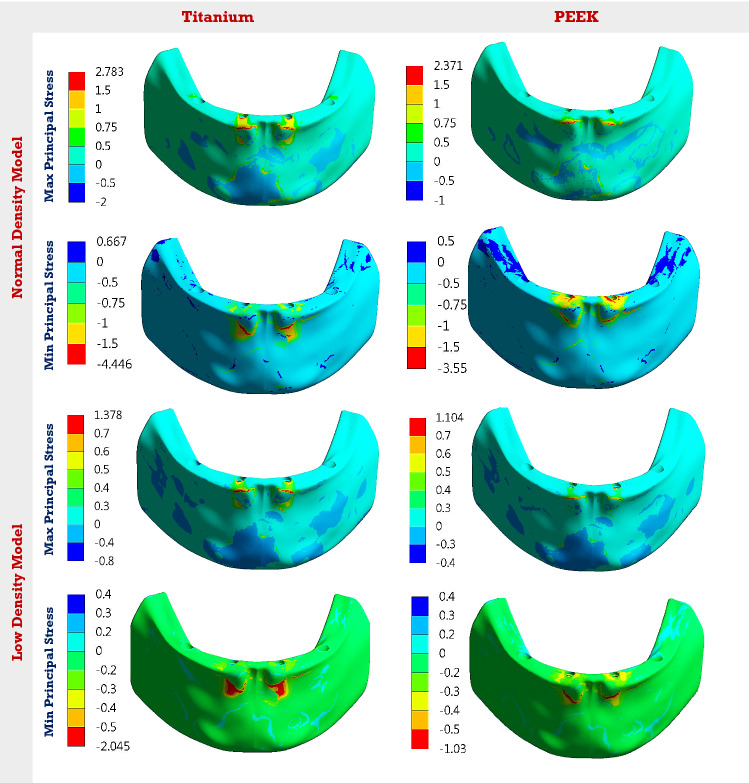
The distribution of max and min principal stresses (MPa) on the spongy bone, under anterior force. Using Ti and PEEK. In the normal and low densities models

Maximum and minimum principal stresses and strains were extracted to analyze the tensile and compressive patterns on bone tissues. Tables 4 and 5 illustrate the peak max and min principal stresses and strains of cortical and spongy bones, under the three mastication forces, in normal and low densities models. Figure 6 presents the distribution of maximum and minimum principal stresses on spongy bone, under the anterior force, using Ti and PEEK materials.

**Table 4:** Peak max and min principal stresses and strains on the cortical and spongy bones, in the normal-density model

		Cortical bone				Spongy bone			
		Principal stress (MPa)		Principal strain (µε)		Principal stress (MPa)		Principal strain (µε)	
		Max	Min	Max	Min	Max	Min	Max	Min
Uni-lateral	Ti	18.05	−44.24	1410	−2625	2.75	−4.23	2192	−2457
	PEEK	10.22	−39.30	1069	−2375	1.37	−2.67	1268	−2063
Bi-lateral	Ti	16.79	−52.27	1299	−2873	2.93	−4.23	2304	−2460
	PEEK	10.73	−36.83	994	−2112	1.43	−2.78	1154	−1901
Anterior	Ti	36.75	−45.53	1933	−2742	2.78	−4.44	1839	−2667
	PEEK	19.90	−37.37	1500	−2250	2.37	−3.55	1390	−2299

**Table 5:** Peak max and min principal stresses and strains on the cortical and spongy bones, in the low-density model

		Cortical bone				Spongy bone			
		Principal stress (MPa)		Principal strain (µε)		Principal stress (MPa)		Principal strain (µε)	
		Max	Min	Max	Min	Max	Min	Max	Min
Uni-lateral	Ti	40.14	−73.26	2315	−4289	1.849	−2.194	6499	−6709
	PEEK	13.76	−55.83	1544	−3386	1.320	−1.100	3375	−3968
Bi-lateral	Ti	37.36	−71.11	2163	−3871	1.84	−2.143	6599	−6749
	PEEK	14.77	−48.75	1390	−2935	1.36	−1.070	3373	−3750
Anterior	Ti	39.06	−66.05	2029	−3923	1.378	−2.045	5126	−6619
	PEEK	10.70	−51.55	1228	−3056	1.10	−1.030	3521	−4020

In the normal-density model, under unilateral force, the peak max and min principal stresses were reduced by (43.37 and 11.16%) for cortical bone, and (50.18 and 36.87%) for spongy bone, using PEEK, compared to titanium. These values were changed to (36.09 and 29.53%) and (51.19 and 34.27%) for cortical and spongy bones, respectively, under bilateral force. In anterior force, the reduction in peak max and min principal stresses were (45.85 and 17.92%) for cortical bone and (14.74 and 20.04%) for spongy bone, using PEEK material. For cortical bone, using PEEK, the peak max and min principal strains were reduced by (24.18 and 9.52%), (23.47 and 26.48%), and (22.4 and 17.94%) under unilateral, bilateral, and anterior forces, respectively. For spongy bone, the values of peak max and min principal strains were reduced by (42.15 and 16.03%), (49.91 and 22.72%), and (24.41 and 13.79%) under the three forces, respectively.

In the low-density model, under unilateral force, the peak max and min principal stresses were reduced by (65.71 and 23.79%) for cortical bone, and (28.61 and 49.86%) for spongy bone, using PEEK instead of titanium. Under bilateral and anterior forces, the peak max and min principal stresses were reduced by (60.46 and 31.44%) and (72.60 and 21.95%) for cortical bone, and (26.08 and 50.06%) and (20.17 and 49.63%) for spongy bone. The peak max and min principal strains for cortical bone were reduced by (33.30 and 21%), (35.73 and 24.17%), and (39.47 and 22.10%) under unilateral, bilateral, and anterior forces, using PEEK. For spongy bone, the values were changed to (48.06 and 40.85%), (48.88 and 44.43%), and (31.31 and 39.26%), respectively.

## Discussion

The purpose of this research was to conduct in vitro stress–strain analysis to investigate the possibility of using PEEK material, in place of titanium in the fabrication of “All on four” prosthesis, in different bone models. A 300 N vertical force was applied in three positions to stimulate the different mastication mechanisms. The max von Mises stresses were extracted for all prosthetic components due to their ductile properties. The maximum and minimum principal stresses and strains were computed for the cortical and spongy bones, due to their brittle and ductile properties.

The stress–strain analysis is a branch of engineering that uses a variety of techniques to determine the stresses and strains in materials and structures that have been subjected to forces. There are many techniques of stress–strain analysis used in dental research; among them the photoelastic technique, digital image correlation technique, electrical resistance strain gauge, and finite-element method (FEM) [31, 43–45]. The finite-element method (FEM) is a numerical method of analyzing the stresses and strains at any point. In dentistry, it offers several advantages over other methods, including the ability to accurately depict complex geometries, apply modifications, propose new designs, define multiple boundary conditions, stimulate different materials, and then extract the internal stresses and strains at any point within a short calculation time [31]. Hence, the finite-element method has been used in many dental studies such as [15, 24, 26, 29–31, 34, 37, 39, 40].

Malo et al. [46] introduced the “All on- four” technique, which uses four implants with distal implants inclined 30 or 45º to reduce the cantilever length and enhance the stress distribution on the implants system. The prosthetic parts in “All on four” have been fabricated from metallic materials such as titanium. The limitations of metals have encouraged researchers to look for alternative polymeric materials (e.g., PEEK). PEEK is a non-allergic, radiolucent, biocompatible material with great thermal stability, good mechanical, thermal and chemical properties, and low plaque affinity [18–21]. In addition, the mechanical properties of PEEK remain unchanged while being sterilized with steam, gamma radiation, and ethylene oxide [47]. PEEK properties can be improved to suit the biological demand by adding other materials such as glass fibers (GFR-PEEK), carbon fibers (CFR-PEEK), or ceramic fillers (Bio-HPP). All PEEK compounds are characterized by good strength, inertness, resistance to chemical erosion, compatibility with image techniques, good esthetic appearance, and biocompatibility [20–22].

Titanium has been used in implantology due to its excellent properties. However, it has limitations such as hypersensitivity, allergic reactions, casting problems, porosity, and peri-implantitis related surface corrosion. In addition, the elastic modulus mismatch between titanium and bone has caused bone overloading, which has a major issue for implant stability [5–8]. Researchers have predicted that these negative aspects would be reduced with the use of PEEK implants [22–24]. The properties of PEEK implants can be modified by the addition of other materials or by surface treatment, to enhance cell adhesion, proliferation, biocompatibility, and osteoconductive properties [20–23].

In the fabrication of the abutments, various metallic and ceramics materials such as titanium, gold, and zirconia have been used. As the surface of the abutment is extremely vulnerable to the subgingival formation of plaque/biofilm, the abutment material should produce less biofilm accumulation on its surface. The limitations of titanium abutments were represented in their dark color, the hypersensitivity reactions, and the formation of biofilm [5, 12]. While the limitations of zirconium abutments were represented in their high elastic modulus and density [48, 49]. In comparison to titanium and zirconia, PEEK material has good mechanical properties and can produce less biofilm accumulation, making it suitable for the fabrication of abutments [20, 25].

Standard bars in hybrid prostheses have been made from metals such as titanium and gold, because of their rigidity, strength, and biocompatibility. Metals, however, have drawbacks that led the researchers to look for alternative materials. An esthetic alternative was a zirconia bar, due to its rigidity, durability, and good mechanical and chemical properties. As rigid bars may help in transferring fewer stresses to the prosthetic parts, implants, and bone, avoiding prosthesis failure, according to some researchers [13, 50, 51]. Researchers, with another view, recommended the usage of semi-rigid and soft bars, to evenly distribute the load and hence dampen the stresses transmitted to the bone [15, 26].

In this paper, the null hypothesis—assuming that the polymeric PEEK material would exhibit the lowest stresses and strains in bone tissues and represent the best scenario, in contrast to titanium—was accepted. Using PEEK instead of titanium in the fabrication of “All on four” parts, the stresses generated on bar, implants, and multi-unit abutments were reduced in all bone models, under the three different forces. Moreover, the stresses and strains on cortical and spongy bones were significantly reduced. However, the stresses on acrylic denture and mucosa were increased. These findings were in close agreement with the studies [15, 19, 26, 40, 52–55].

In Mohammed thesis [40], the stress distribution in overdenture mandibular prosthesis was evaluated using PEEK implants in place of titanium. The results demonstrated that the PEEK implants reduced the max von Mises stresses on cortical and spongy bones by 13 and 46%, respectively, however, increased the mucosal stress. Haseeb et al. [52] compared the stress distribution and deformation in the bone surrounding the implant, using three different implant biomaterials (Titanium, Zirconia and PEEK), under vertical and oblique loads. The results illustrated that PEEK can be used as an alternative implant biomaterial to titanium.

In Tekin et al. research [19], the stress generated in the peri‑implant bone was compared using PEEK abutment as an alternative to titanium, under the PEEK crown. In comparison to titanium, PEEK abutment caused a reduction of 1.1% on the max von Mises stress on the bone. In addition, Korsel [53] applied a 3D finite analysis to evaluate the stress distributions in implants, screws, and bone, using different abutment materials, among them the modified PEEK (BIOHPP). The result illustrated that BIOHPP abutment reduced the stresses on implant and bone by 10.9 and 15%, respectively, however, increased the screw stress.

In the fabrication of the bar, Malo et al. [54] illustrated that PEEK-acrylic resin prostheses might present a viable treatment option for edentulous patients, but longer-term validation is still needed. In Haroun et al. research [15], the maximum and minimum principal stresses on bone were extracted using PEEK and titanium bars, on maxillary prostheses utilized “All on four” concept, and ceramic superstructure with zirconium. The results demonstrated that the PEEK bar reduced the maximum and minimum stresses on bone by 32.3 and 41.9%, respectively, when the force was delivered from the opposing acrylic All-on-4 prosthesis. In Shash et al. research [55], a 3D model of a mandible with a hybrid prosthesis was constructed and stimulated with different bar materials (Titanium, CFP 30 and 60%, BIOHPP, PEKK and PEEK). The simulation results clarified that the PEEK bar reduced the stresses on cortical and spongy bones by 3.44 and 3%, however, increased the mucosal stress. Moreover, Chen et al. [26] applied a finite-element analysis of mechanical function for four designs of removable partial denture using three framework materials (CoCr, Ti-6Al-4 V alloy and PEEK). The results illustrated that the PEEK framework produced the lowest stress on periodontal ligament, the highest stress on the mucosa, and the lowest stress on the framework, compared with CoCr and Ti–6Al–4V alloy.

This paper also investigated the influence of bone density on the selection of “All on four” material. The results illustrated that the density of spongy bone influenced the stresses and strains generated on all parts especially the mucosa, spongy and cortical bones. The decrease in the density of spongy bone decreased the stress on it, and consequently increased the stresses on cortical bone and mucosa. In addition, the low-density model exhibited high maximum and minimum principal strains on cortical and cancellous bones, compared to the normal-density model.

From the extraction of results, using titanium “All on four”, the max von Mises stresses of all prosthetic components (bar, copings, screws, abutments and implants) did not exceed the yield strength of titanium (~ 900 MPa [38]). Likewise, using PEEK material, the max von Mises stresses of all prosthetic components did not exceed the yield strength of PEEK (140-170 MPa [19, 20]). Therefore, no damage or breakage might occur in all prosthetic parts, under 300 N static force. The results also clarified that PEEK material increased the stress value of mucosa, in the two bone models. However, this value was lower than the pain threshold value (0.63–1.2 MPa [56]); hence, no mucosal pain or inflammation might occur.

The maximum and minimum principal stresses were extracted for cortical and spongy bones, then compared with the tensile and compressive yield strengths, following the failure theory of principal stress [57]. For cortical bone, with the normal-density, the tensile and compressive yield strengths were (100 and 140 MPa [58]). Using a safety factor of 1.5, the permissible limits were nearly (66 and 93 MPa), respectively. For spongy bone, the permissible tensile and compressive limits were approximately (2 and 2.33 MPa) and (6.5 and 10.5 MPa) for low and normal densities [58, 59]. From Tables 4 and 5, the maximum and minimum principal stresses of cortical bone did not exceed the permissible limits in all cases. For spongy bones, the maximum and minimum principal stresses were far from the permissible limits in the normal-density model. In the low-density model, the stresses approached the limits using titanium. Therefore, PEEK material was recommended to be used in the fabrication of “All on four” parts in this case.

Excessive strain might cause damage to the implant–bone interfaces, causing implant loss. Consequently, the maximum and minimum principal strains were also extracted for the cortical and spongy bones and compared with the critical limits. The damage of cortical bone occurred when the strain exceeded 2500 με in tension and 4000–5000 με in compression [30]. For spongy bone, the limits were 7000–8000 με in tension and compression [60]. The results of the present study demonstrated that in the normal-density model, the maximum and minimum principal strains of cortical and spongy bones did not exceed the critical limits, using titanium and PEEK materials. In the low-density model, PEEK material was preferred as it reduced the strains of bone tissues.

From the evaluation of results, PEEK material can be used in the fabrication of “All on four” prosthetic parts, in place of titanium. In vivo, PEEK material is expected to offer several major benefits over titanium, including improved performance and esthetics, better design freedom, fabrication of lighter prostheses, reduced overall system cost, and reduced fabrication issues and the risk of mechanical problems. In addition to overcoming the allergic and hypersensitivity reactions generated by titanium implants, and reducing the formation of biofilm/plaque generated on titanium abutments. In comparison to titanium, according to the results, PEEK material is preferred for usage in low-density mandibles because it minimizes the stresses and strains on bone tissues, lowering the possibility of bone loss and increasing the prosthesis stability. However, additional investigations and long-term studies on PEEK material are needed in near future to avoid its disadvantages.

The main disadvantage of PEEK material in prosthetic dentistry is its low surface energy [20–23]. The biological inertia of PEEK makes the bone integration between the PEEK implant and the host bone tissue weak and may clinically encounter complications (e.g., implant displacement) which lead to unsatisfactory results in vivo. Hence, considerable efforts should focus on modifying the surfaces of PEEK implants to enhance the ingrowth of osteoblasts (osteoconduction), direct contact with surrounding bone (osseointegration), and stimulation of immature cells into osteogenic cells (osteoinduction).

This finite-element model had limitations, such as the homogeneities and the isotropic linear elasticity of the material properties. Altering the mandibular properties with the anisotropic assumption may result in different stress and strain distributions. The bone–implant and implant–abutment interfaces were also assumed to be completely bonded, and this may not accurately reflect the actual clinical situation. In addition, the applied load was static, although the bone tissues respond to the dynamic loads. Hence, this finite-element analysis may not accurately mimic the true clinical scenario.

## Conclusion

Within the limitations of this study, the followings were concluded:

- The usage of PEEK in the fabrication of “All on four” parts reduced the stresses and strains generated on the cortical and spongy bones, and increased the mucosal stress, unlike titanium.
- The density of spongy bone influenced the choice of “All on four” material. In the low-density model, PEEK material was preferred to reduce the stresses and strains generated on different bone tissues.
- Further research and many controlled clinical trials on PEEK implants and abutments are required in near future.

## Data Availability

All data generated or analyzed during this study are included in this published article.
